# Condition Monitoring Using a Latent Space of Variational Autoencoder Trained Only on a Healthy Machine

**DOI:** 10.3390/s24216825

**Published:** 2024-10-24

**Authors:** Iwona Komorska, Andrzej Puchalski

**Affiliations:** Faculty of Mechanical Engineering, Casimir Pulaski Radom University, 26-600 Radom, Poland; andrzej.puchalski@urad.edu.pl

**Keywords:** condition monitoring, diagnostic signal, variational autoencoder, latent space, feature extraction, dimensionality reduction, t-SNE method

## Abstract

Machine learning generative models have opened up a new perspective for automated machine diagnostics. These methods improve decision-making by extracting features, classifying, and creating new observations using deep neural networks. Generative modeling aims to determine the joint distribution of input data. This contrasts traditional methods used in diagnostics based on discriminative models and the conditional probability distribution of the target variable at known feature values. In the variational autoencoder (VAE) algorithms trained by the authors, the parameters of diagnostic features are random variables, the distributions of which can be approximated based on data, and the identification of probability distributions is based on variational inference. Variational inference is a tool that deals with difficult statistical problems and is usually faster than classical methods. VAEs can detect anomalies, predict failures, and optimize processes. This paper proposes an unsupervised approach to fault diagnosis using only healthy data with automatic feature extraction from the continuous probabilistic latent subspace of the VAE encoder and reduction in PCA or t-SNE. The solution, verified in the example of simulation data, is a response to a common problem related to the lack or difficulty of obtaining marked data in defected states of devices and mechanical structures.

## 1. Introduction

In order to implement a proactive maintenance strategy, it is essential to utilize advanced technologies to anticipate equipment failures. One way to achieve this is through the development of artificial intelligence and machine learning-based methods that can reduce the reliance on expert knowledge for traditional monitoring and diagnostic procedures. These methods can automatically carry out data contextualization, feature extraction, and classification using deep neural network models. Unlike humans, this sophisticated analytical approach considers thousands of variables and constraints to enhance the decision-making process. With the development of deep neural networks, there is a growing interest in generative models for specific industrial applications, especially in intelligent solutions for technical diagnostic tasks.

Generative Artificial Intelligence (AGI) represents a different approach compared to traditional ML-based diagnostic classifiers like vector machine (SVM) or K-nearest neighbors (KNNs) [1,2]. Generative models [3] are multilayer neural networks that allow for the approximation of multidimensional probability distributions. Unlike discriminative models, which are based on the conditional probability distribution of the target variable given known feature values, generative modeling aims to determine the joint distribution of input data and create new observations. In generative algorithms, the parameters of diagnostic features are considered as random variables whose distributions can be approximated based on data. The identification of probability distributions is often based on variational inference, which is a tool that addresses challenging statistical problems and is typically faster than classical methods like Markov chain Monte Carlo (MCMC) sampling.

The use of generative deep neural networks in technical diagnostics necessitates proper model selection and careful training on relevant data. Ever since the initial publications on variational autoencoders (VAEs) [4,5,6] and generative adversarial networks (GANs) [7,8,9], these models have been increasingly used in the detection of faults in mechanical devices and structures. A comprehensive review of deep generative machine learning models utilized in engineering design from 2014 to 2021 is outlined in [10]. In a study cited as reference [11], researchers utilized the deconvoluted data representations present in the latent space of autoencoders. They utilized the reconstruction error as an indicator of machine health. The experiment involved testing the data on three publicly available degradation datasets of turbofan engines, each with different operating conditions and failure patterns. Additionally, tool wear data from a milling machine operating under varying conditions were taken into account. A deep autoencoder (DAE) and a variational autoencoder were both employed in this analysis. The authors of [12] proposed using the total loss in the unsupervised learning of a variational autoencoder as an indicator of outliers in IoT sensor databases. The Remaining Useful Life (RUL) of rolling bearings is crucial for assessing the safety and reliability of rotating machinery. In a proposed method for constructing the Health Index to quantify degradation states and RUL using a CAE model and mapping function, latent variables in the hidden layer of the autoencoder were sampled, and a multidimensional state mapping function based on dimensionality reduction and Euclidean distance statistics was used [13]. Additionally, one paper [14] presents a similarity-based curve fitting method for estimating the RUL of a mechanical system using an autoencoder scheme based on a bidirectional recurrent neural network (RNN).

In reference [15], a method for diagnosing issues with rotating machines was introduced. This method does not rely on data from the damaged state, and it operates under variable load and temperature conditions. It utilizes order analysis and a deep neural network to establish connections between amplitude values in the order spectrum and factors such as rotational speed, oil temperature, and current. The network is trained using data from normal, failure-free operating conditions. The method proposes a diagnostic parameter that can identify damages based on the principles of classical spectral vibroacoustic diagnostics. In one paper [16], a strategy based on generative VAE and GAN models was proposed for constructing a deep digital twin (DDT) of a real asset using health sensor data and for automatically generating the Health Index. The monitoring strategy presented in reference [17] is based on the reconstruction error, which serves as a health indicator. This is derived from the DAE + LSTM model trained using simulated high-dimensional monitoring time series of aero-engine performance in the operational state. In reference [18], a semisupervised LSTM-based VAE-GAN model was utilized to address the issue of small and noisy signals in wind turbines.

The VAE’s generative properties were utilized in [19] to augment the ultrasonic-guided wave signals in the composite panel within the training datasets for varying temperature conditions. Ongoing research is focusing on applying supervised and unsupervised machine learning methods to structural health monitoring (SHM). In a recent study [20], a new approach using ultrasonic Lamb waves and a DAE was proposed for effectively detecting and classifying fatigue damage in composite plate structures. In the paper [21], two unsupervised data-driven approaches for fault localization using ultrasonic guided waves are presented. These approaches utilize convolutional autoencoders (CAEs) and conditional generative adversarial networks (CGANs). An unsupervised learning pre-trained convolutional autoencoder (TL-CAE) capable of detecting and localizing damage that is not visible during training is presented in reference [22]. This autoencoder can perform structural health monitoring without any preprocessing or knowledge of material properties, by training on real signals from undamaged material.

In a study described in reference [23], a CAE was trained using only data from healthy conditions. The CAE was used to detect structural damage by comparing compressed data from damaged conditions with compressed data from healthy conditions. The main goal of the study in reference [24] was to identify structural damage in bridges under variable loads without needing reference data. This was achieved by using a VAE trained on real-world vibration acceleration signals. This paper discusses the use of VAE models for various purposes such as generating failure scenarios, anomaly detection, failure prediction, and process optimization [25,26]. A variational autoencoder combines Bayesian probability theory and deep learning. The VAE encoder maps input data to a continuous probabilistic latent subspace, and the VAE decoder generates output data by randomly sampling in the continuous latent subspace and decoding these samples.

This paper introduces a unique approach to fault detection in rotating machines. Unlike traditional methods that require the manual selection of diagnostic features, this approach uses an unsupervised, data-driven technique that relies solely on healthy data. A key innovation is the analysis of data from a continuous observation subspace, which is encoded as a multidimensional normal distribution in the latent space using mean value vectors (μ) and standard deviation (σ). This procedure, combined with the nonlinear feature reduction method, allows for automatic probabilistic feature extraction, significantly reducing the subjectivity involved in selecting diagnostic features. Additionally, this paper introduces new distance measures for diagnostic feature vectors.

A common problem in machine diagnostics is the lack of data for fault states. Unbalanced databases, especially those with a lack of data from damaged objects, pose significant challenges in data analysis and machine learning. When the majority of the data represent nondamaged objects, machine learning models tend to learn more about these and less about the minority class (damaged objects). This can lead to overfitting, where the model performs well on the majority class but poorly on the minority class. The model may not learn the critical features that distinguish damaged objects due to their underrepresentation in the training data. One solution may be to augment data from the faulted object. However, in this article, a different approach is taken. This enables an effective monitoring and diagnosis of the system’s current state based on the learned features only from the healthy state.

This paper is structured with an introduction and three chapters. Section 2 discusses the operation of the VAE model, the method of reducing the feature vector, and the specifications of the databases used. Section 3 elaborates on the individual stages of the analysis of the latent vector of diagnostic features leading to obtaining the optimal Fault Index indicator, while conclusions and further research plans are presented in Section 4.

## 2. Materials and Methods

### 2.1. Variational Autoencoder (VAE)

In the era of the Industrial Internet of Things (IIoT) and big data, physical modeling in technical diagnostics is replacing empirical modeling using AI and ML tools. The next step is to move away from the manual selection of diagnostic features to automatic selection. Instead of the conditional probability distribution of the target variable given known feature values, which was the essence of discriminative models, the challenge of generative models is to approximate the joint distribution of input data.

This paper discusses a VAE model [4,5,6,26], which is a type of neural network comprising an encoder and a decoder. The VAE transforms real data into a Gaussian distribution using a probabilistic encoder (p(z|x)), ensuring the latent space is normalized to standard normal distributions through a regularization term represented by the Kulback–Leibler (KL) divergence. The Gaussian distribution is then passed to a probabilistic decoder (p(x|z)) to generate synthetic data. The network is optimized by backpropagating the reconstruction error. The loss function is a combination of the reconstruction loss and the KL divergence. In contrast to standard autoencoders, the VAE’s diagnostic feature subspace consists of a multidimensional probability distribution, allowing for the flexible generation of new samples. The VAE’s use of a probabilistic Gaussian distribution enables a flexible and probabilistic modeling of the data representation, facilitating the generation of new samples.

### 2.2. Feature Vector Reduction Methods

#### 2.2.1. Principal Component Analysis (PCA)

PCA is a statistical technique that simplifies complex datasets by transforming the data into a new set of variables called principal components. These components are ordered so that the first few retain most of the variation present in the original dataset. Essentially, PCA helps reduce the dimensionality of the data while preserving as much information as possible [27,28].

PCA works by decomposing a covariance matrix into its eigenvectors. Covariance measures the degree of the relationship between different variables in multivariate data. In the first phase of the PCA method, the mean value of the gathered data is subtracted. Then, the covariance matrix is created, and the eigenvalues and eigenvectors of the covariance matrix are computed. Furthermore, the cumulative energy content of each eigenvector is calculated. In the following stage, the eigenvectors are sorted from the largest one to generate an ordered orthogonal basis, allowing for the expression of the data in terms of only a few orthogonal basis vectors rather than all the covariance matrix eigenvectors.

#### 2.2.2. t-Distributed Stochastic Neighbor Embedding (t-SNE)

The t-SNE method is a technique used to reduce the dimensionality of high-dimensional data, making them easier to visualize and understand. It calculates the similarities between data points in the high-dimensional space and maps them to a lower-dimensional space, typically 2D or 3D, while preserving the structure and relationships between the points [29].

The algorithm begins by measuring the likelihood of points being neighbors in the high-dimensional space and then adjusts the positions of points in the low-dimensional space to minimize the difference between the two spaces. This process helps to capture nonlinear relationships and often reveals clusters and patterns that are not apparent in the original high-dimensional data.

The choice of distance metric in the t-SNE method is essential for calculating similarities between data points in high-dimensional space. The most commonly used distance metric in t-SNE is the Euclidean distance, which measures the straight-line distance between two points in high-dimensional space to determine their similarity. However, t-SNE can also work with other distance metrics depending on the nature of the data and specific analysis requirements. Some alternative distance metrics include Chebyshev, Mahalanobis, and cosine.

A crucial parameter in the t-SNE method is perplexity, which influences how the algorithm balances attention between the local and global aspects of the data. Perplexity can be thought of as a measure of the effective number of neighbors each point has. Essentially, perplexity controls the trade-off between focusing on the local data structure (small perplexity values) and capturing the broader, global data structure (large perplexity values). Typical values for perplexity range from 5 to 50. Adjusting this parameter can significantly affect the resulting visualization, with different perplexity values highlighting different aspects of the data.

### 2.3. Proposed Method

A new two-stage architecture for machine learning analysis was proposed. This architecture has the ability to accurately predict faults using only the machine operating signals in a healthy state, in the frequency domain, without the need for a complex preprocessing and preselection of spectral features.

**Figure 1:**
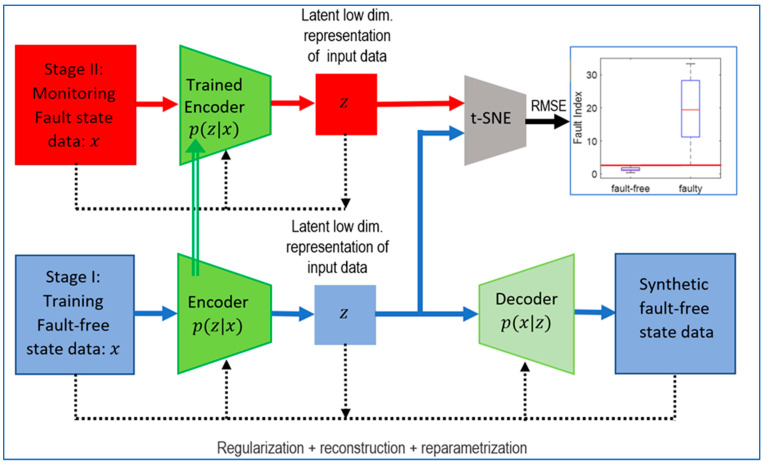
Latent low-dimension vector error method.

In the first stage, a VAE autoencoder is used to automatically select reference diagnostic features. During the training of the network, a multidimensional hidden vector (mi, sigma) of statistical features is generated based on the representation of data in the healthy state. During the monitoring stage, samples representative of machine faults are passed through only the encoder of the trained VAE network, and the generated hidden vector is then compared with the vector obtained during network training (Figure 1).

Due to the linear dependence of the hidden vector features, the dimensionality is reduced in the second stage. The Fault Index based on the RMSE uses vector distance measures. For this purpose, PCA and t-SNE transformations are employed to reduce dimensions.

### 2.4. Simulation Data

The method was demonstrated using simulation data from the triplex reciprocating pump model, which is available in Matlab R2024a [30]. Three types of faults were simulated in the pump: leaking pump cylinder, blocked pump inlet, and increased pump bearing friction. The measurements include conditions in which no, one, or some faults occur. In this example, 5 operating states were used: fault-free, seal leakage, blocked inlet, worn bearing, and mixed failures of seal leakage and blocked inlet states. The description of the faults and their exact specifications can be found in [31]. The model accounts for noise, so each simulation with the same error parameters differs from the previous one. In [32], fault diagnostics were performed based on classical feature extraction from the pump operating signals, selected by the expert system. The approach proposed in this method is based on automatic feature extraction by the VAE network and a further reduction in the feature vector. The network is trained only on healthy cases.

**Figure 2:**
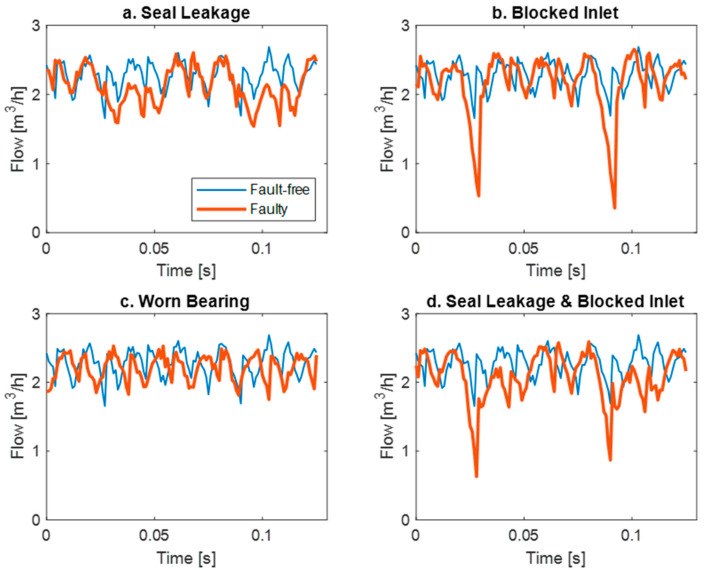
The flow waveforms for failed states in comparison to the fault-free signal for different faults.

The operating signal from the pump flow is utilized for diagnostic purposes. Figure 2 illustrates the flow signal for failed states in comparison to the fault-free signal.

The pump motor speed is 950 rpm, which means the pump cycle time is 0.063 s. Since the pump has 3 cylinders, the flow is expected to change at a frequency of 3 times 15.833 Hz, which equals 47.5 Hz. The sampling frequency is 1000 Hz.

**Figure 3:**
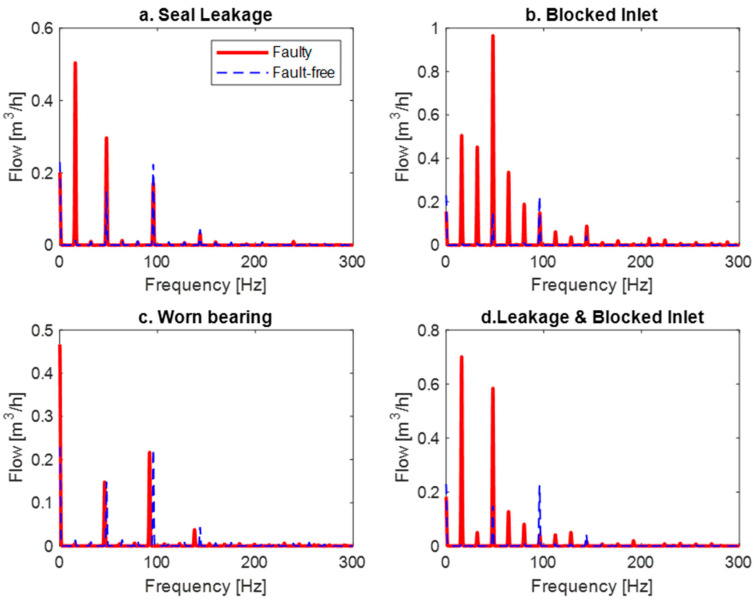
The flow frequency spectra for failed states in comparison to the fault-free signal for different faults.

Figure 3 shows the frequency spectra of the flow signal, compared to the fault-free state and the faults for the previously mentioned faults.

## 3. Results and Discussion

The data were represented as a frequency spectrum and then fed into a variational autoencoder (VAE) consisting of two hidden layers. The encoder and decoder are almost symmetrical, with the only difference being the last layer of the encoder, which produces a latent vector representing the mean and standard deviation of the input signal features. A 16-dimensional arbitrary vector was chosen for the latent space, which holds the compressed form of the input image in a reduced-dimensional latent space. The data were initially input into the encoder as a frequency spectrum and then reshaped into a 28 × 28 matrix. Hidden layers (convolutional and ReLU) were used, and a fully connected layer with twice as many output channels as the number of hidden channels was utilized to obtain a combined vector of means and variances.

**Figure 4:**
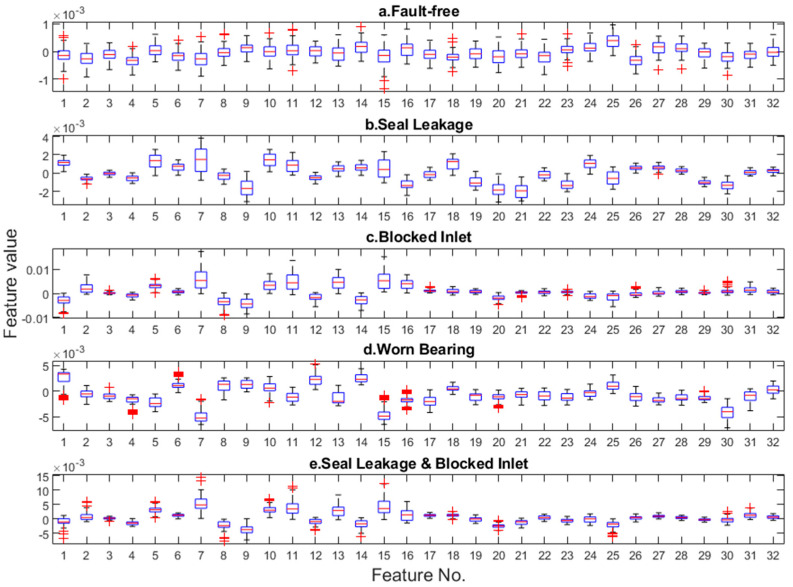
Latent vector for healthy pump and four types of faults.

The generator takes an input vector drawn from a normal distribution and produces a 2D output with the same dimensions as the original training data. The VAE encoder extracts features from the entered spectra. Figure 4 shows the distributions of these features for individual operating states.

The boxplots for fault-free features are more regular and oscillate near zero compared to the faulty ones.

The Fault Index ${FI}_{VAE}$ is defined using the mean square error between each feature for different operating states ${feat}_{ij}$ and the mean value of each feature in the healthy state $\bar{{feat}_{i0}}$

(1) $${FI}_{VAE}=RMSE({feat}_{ij},\bar{{feat}_{i0}}),$$

where i, j denote the feature number and operational condition, respectively, and take the values i=1…32, j=0…4.

**Figure 5:**
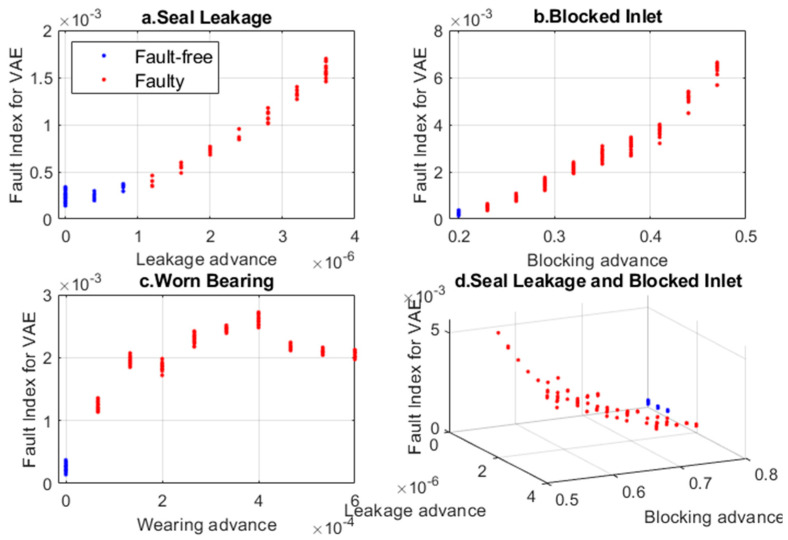
Comparison of Fault Index calculated based on all features extracted by VAE for different operating states.

The change in the Fault Index for various developments of faults is illustrated in Figure 5.

As the failure advancement increases, the indicator also increases, although for the ‘Worn Bearing’ state, it does not follow a monotonic change.

**Figure 6:**
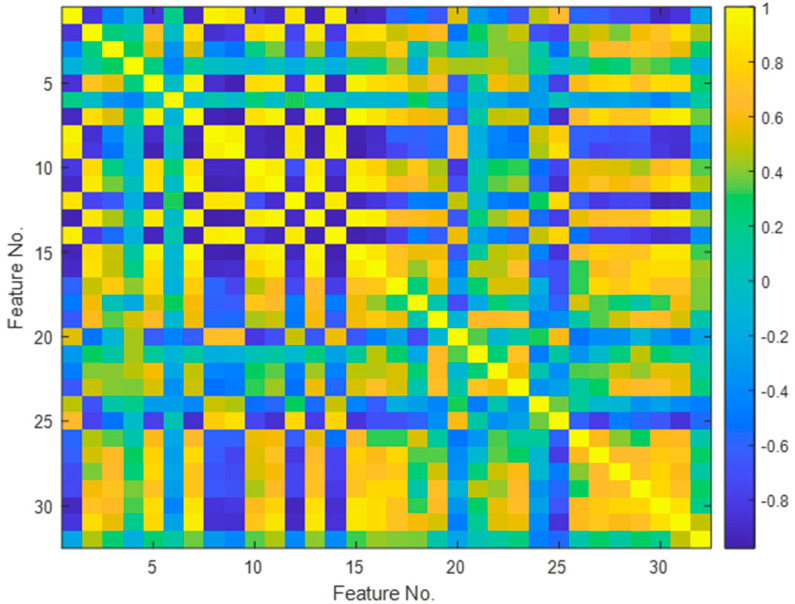
Matrix of correlation coefficients for feature vector.

The mutual correlation between the extracted features was further investigated, and the results are presented in Figure 6.

The characteristics that compose the hidden vector are linearly related to one another and are represented by the yellow squares. Correlated features often contain similar information. Dimensionality reduction helps eliminate redundant data, simplifying the model and reducing the risk of overfitting. Having fewer features also means that there are fewer data to process, speeding up model training and reducing memory requirements. Removing correlated features can enhance model quality by reducing noise and collinearity, leading to more stable and accurate predictions. Additionally, dimensionality reduction facilitates data visualization, which is particularly useful for exploratory analysis and presenting results.

Hence, in the next stage, the feature vector was further reduced. The linear PCA method was initially chosen for its ease of understanding and implementation. It requires fewer computational resources, making it faster than more complex nonlinear methods. The PCA results are deterministic, meaning that the same input data always produce the same results. The PCA results are also easy to interpret because PCA transforms the data into new axes (principal components), which are linear combinations of the original features. These properties make PCA one of the most commonly used dimensionality reduction methods. In many cases, the use of PCA yields satisfactory results, so it is considered the starting method in this paper.

**Figure 7:**
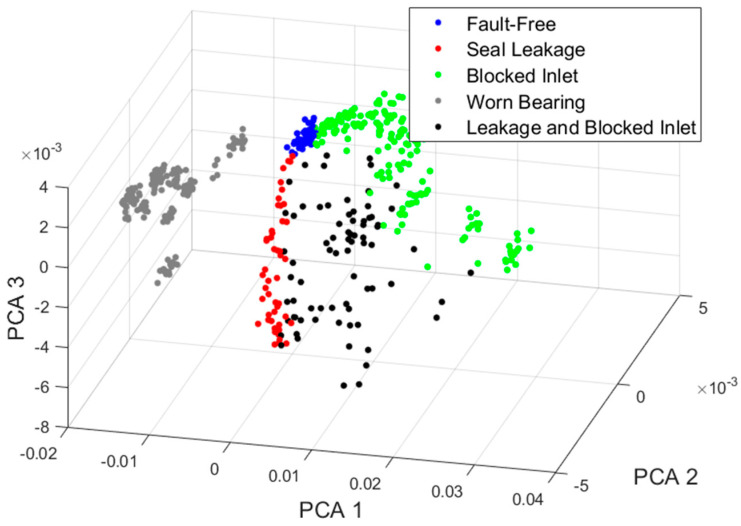
The visualization of the data representation in the space of the first three principal components.

Figure 7 illustrates the initial three components of a healthy pump and different types of damage. These first three components account for 93.66% of all variations.

**Figure 8:**
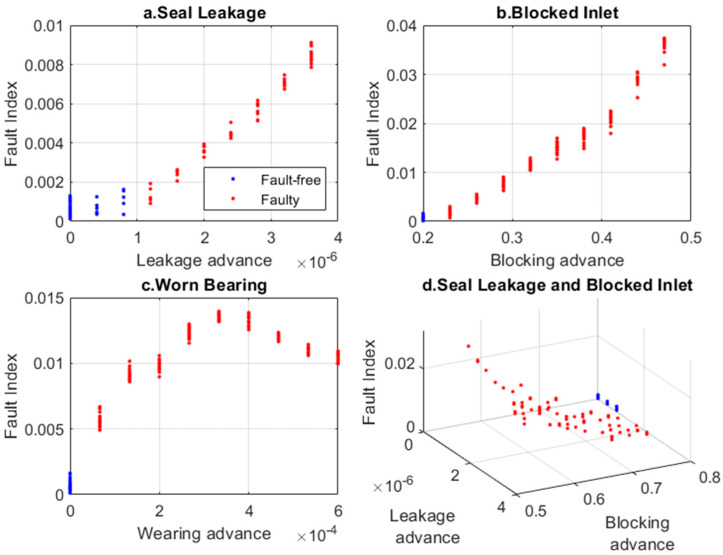
Fault Index for various faults determined based on VAE latent space and PCA.

The set of points representing the healthy pump is the most concentrated. As the damage increases, the points move away from this set, as shown in Figure 8. The set for the ‘Worn Bearing’ condition is the most separated, as are other single damages. However, as expected, the double damage covers a wider area between them.

Figure 8 compares the Fault Index obtained for the PCA-reduced hidden feature vector of the VAE encoder.

(2) $${FI}_{VAE+PCA}=RMSE({PCA}_{ij},\bar{{PCA}_{i0}}),$$

where i, j denote the component number and operational condition, respectively, and take the values i=1…3, j=0…4. $\bar{{PCA}_{i,0}}$ denotes the mean value of each principal component in the healthy state.

**Figure 9:**
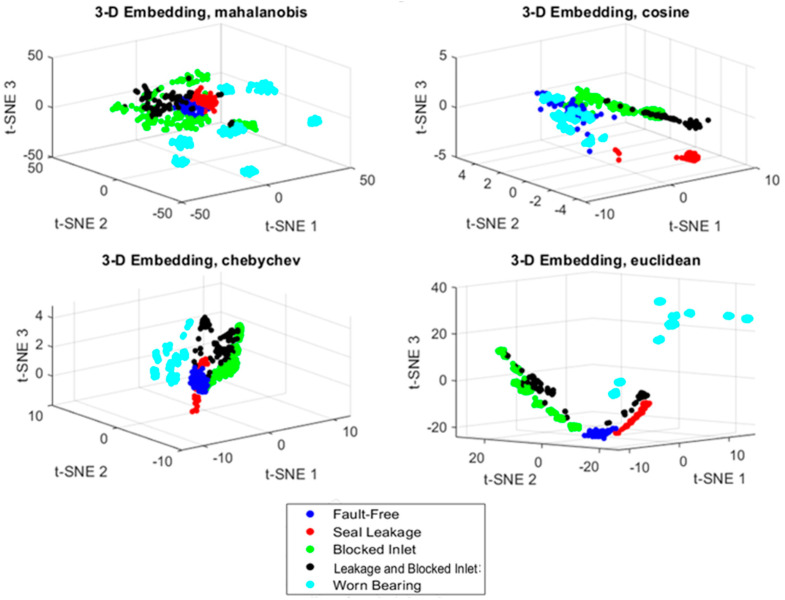
The visualization of the data representation in the space of the first three t-SNE components.

The use of the PCA method for reducing the feature vectors did not have a significant impact on the Fault Index results. The results were similar to the previous ones (compare Figure 5 and Figure 8). In the next step, the feature vector reduction was carried out using the nonlinear t-SNE method, with four distance definitions: Euclidean, Mahalanobis, Chebyshev, and cosine. The most commonly used is the Euclidean distance, and as shown in Figure 9, it best separates sets of points for different faults.

**Figure 10:**
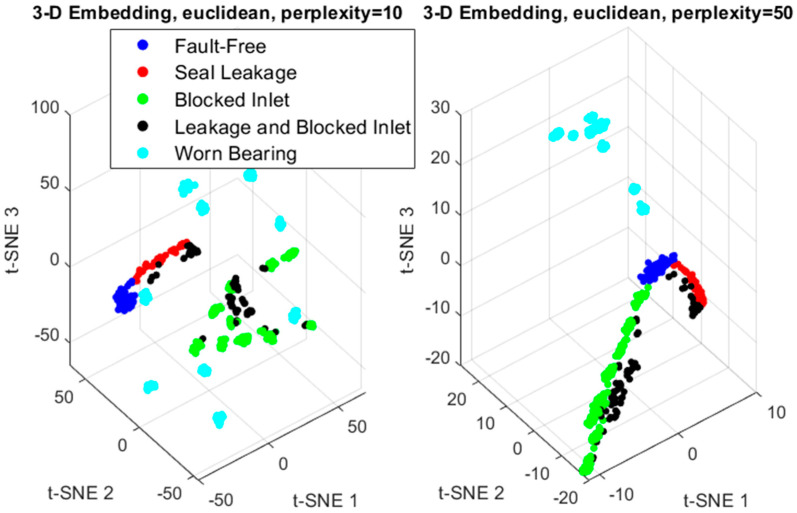
The influence of the perplexity parameter on the clustering of sets using the t-SNE method.

The next parameter to consider is perplexity, which represents the effective number of local neighbors for each data point and is specified as a positive scalar. Typical perplexity values range from 5 to 50. Figure 10 illustrates the impact of the perplexity parameter on data visualization for the values of 10 and 50.

When the perplexity is higher, t-SNE considers more data points as nearest neighbors. For our analysis, a perplexity of 50 (using Euclidean distance) was selected. Similar to PCA, the Fault Index was defined by reducing the hidden feature vector of the VAE encoder using t-SNE.

(3) $${FI}_{VAE+tSNE}=RMSE({tSNE}_{i,j},\bar{{tSNE}_{i,0}}),$$

where i, j denote the component number and operational condition, respectively, and take the values i=1…3, j=0…4. $\bar{{tSNE}_{i,0}}$ denotes the mean value of each principal component in the healthy state.

**Figure 11:**
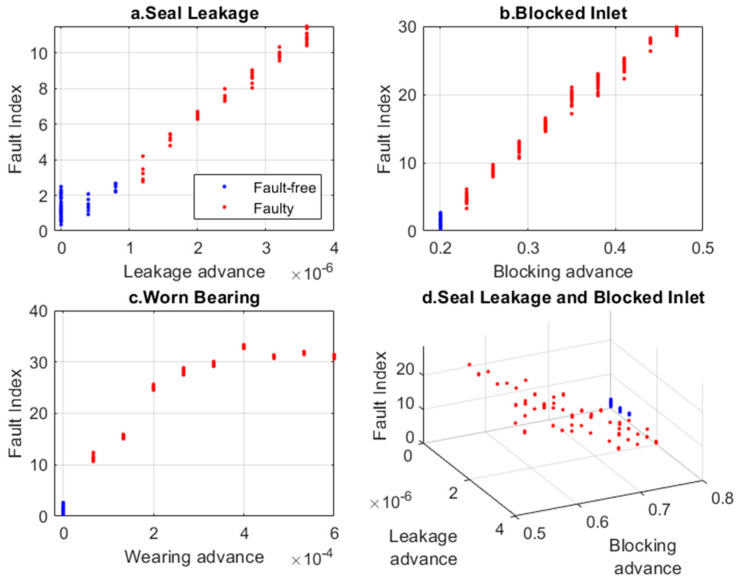
Fault Index for various faults determined based on VAE latent space and t-SNE analysis.

The Fault Index for each operating condition is shown in Figure 11. As the damage increases, the Fault Index for the ‘Seal Leakage’ and ‘Blocked Inlet’ conditions increases linearly, while for the ‘Worn Bearing’ condition, the change is non-decreasing (improved monotonicity).

The method is based on training the variational autoencoder in an unsupervised way using only the signals obtained from the healthy pump. All calculated Fault Index indicators were categorized into two sets: ‘Fault-free’ (healthy) and ‘Faulty’ (regardless of the type of damage). If the maximal Fault Index for ‘Fault-free’ data is exceeded after removing outlier data, this indicates entry into the ‘Faulty’ area.

**Figure 12:**
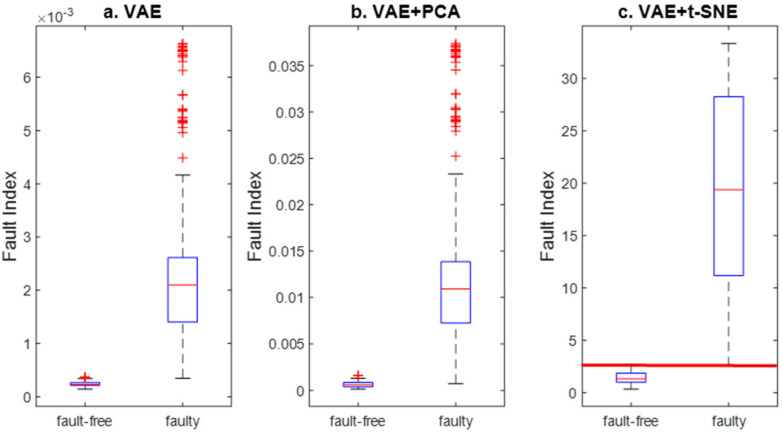
Comparison of Fault Index distributions for investigated methods of feature extraction: (**a**) only VAE, (**b**) VAE and PCA, and (**c**) VAE and t-SNE.

We compared the Fault Index distributions for the ‘Faulty-free’ and ‘Faulty’ states (see Figure 12). Employing PCA to reduce the latent space feature vector did not enhance the separation of the Fault Index ranges. However, using the t-SNE method for the same purpose prevented the Fault Index distributions from overlapping. In Figure 12c, the limit value of the Fault Index indicator is indicated.

Next, important metrics used to evaluate the performance of described models are as follows: Precision, Recall, F1-score, and False Positive Rate (FPR) (Table 1). The Table 1 also includes indicators calculated for the classical model based on the statistical features of signals [32]. Precision is the ratio of true positive predictions to the total predicted positives. Recall (also known as True Positive Rate) measures the ability of the model to identify all relevant instances. The F1-Score is the harmonic mean of Precision and Recall. It provides a single metric that balances both the concerns of Precision and Recall, which is especially useful when you need a balance between the two. The False Positive Rate measures the proportion of actual negatives that are incorrectly identified as positives

The Fault Index calculated for the VAE and t-SNE models indicates the best performance. Slightly inferior results can be achieved by using feature extraction with the VAE latent vector. However, reducing the latent vector features using the linear PCA method leads to worse performance. All automatic feature extraction methods demonstrate better performance compared to traditional methods of selecting statistical features from diagnostic signals.

## 4. Conclusions and Future Work

In this paper, a method for detecting damage using the unsupervised learning of a VAE network trained only for the intact state is proposed. The VAE network has the advantage of being able to generate data that augment the existing database with signals (images) that are similar but not identical, which allows for the feeding of deep neural networks that require a large amount of data, such as CNNs.

A method for the automatic extraction of signal features, which constitute the latent space of the VAE encoder, is proposed. As shown in the example used, the feature vector is linearly correlated and should be reduced. For this purpose, the PCA and t-SNE methods are compared. All automatic feature extraction methods demonstrate better performance compared to traditional methods of selecting statistical features from diagnostic signals.

t-SNE is very good at capturing complex, nonlinear relationships in data that PCA might miss due to its linear nature. This makes t-SNE more effective at preserving the local structure of data and maintaining relative distances between nearby points. Consequently, t-SNE often produces more visually interpretable and meaningful plots, revealing clusters and patterns that may not be apparent with PCA.

Additionally, t-SNE is generally better at handling outliers compared to PCA, which can be significantly affected by them. However, t-SNE is more computationally intensive and requires a careful tuning of parameters like perplexity and learning rate. On the other hand, PCA is faster and less computationally demanding, making it suitable for initial exploratory data analysis when linear relationships are sufficient.

The applications of variational autoencoders in technical diagnostics are being developed alongside large-scale studies on transformer models. These networks utilize an attention mechanism that allows for global feature selection based on long-term dependencies [33]. The authors of this article are also working on using transformers to automatically select diagnostic features based on signals recorded exclusively in the operational state. This aims to enhance the method’s efficiency in terms of accuracy and training.

## Figures and Tables

**Table 1:** Key evaluation metrics for anomaly detection models.

Model	Precision [%]	Recall [%]	F1-Score [%]	FPR [%]
Classical	95.4	95.4	95.4	15.6
VAE	99.5	99.3	99.4	3.3
VAE + PCA	99.1	98.2	98.6	6.6
VAE + t-SNE	**100**	**99.5**	**99.8**	**0**

## Data Availability

Data are contained within the article.
